# Structural and functional analysis of stress-inducible genes and their promoters selected from young oil palm (*Elaeis guineensis*) under salt stress

**DOI:** 10.1186/s12864-022-08926-6

**Published:** 2022-10-31

**Authors:** Thalita Massaro Malheiros Ferreira, Jaire Alves Ferreira Filho, André Pereira Leão, Carlos Antônio Ferreira de Sousa, Manoel Teixeira Jr. Souza

**Affiliations:** 1 Graduate Program of Plant Biotechnology, Federal University of Lavras, 37200-000 Lavras, MG CP 3037, Brazil; 2 Brazilian Agricultural Research Corporation, Embrapa Agroenergy, 70770-901 Brasília, DF Brazil; 3 Brazilian Agricultural Research Corporation, Embrapa Mid-North, 64008-780 Teresina, PI Brazil

**Keywords:** Abiotic stress, Salinity, Transcriptomics, qRT-PCR, RNA-Seq, Strategy

## Abstract

**Background:**

Soil salinity is a problem in more than 100 countries across all continents. It is one of the abiotic stress that threatens agriculture the most, negatively affecting crops and reducing productivity. Transcriptomics is a technology applied to characterize the transcriptome in a cell, tissue, or organism at a given time via RNA-Seq, also known as full-transcriptome shotgun sequencing. This technology allows the identification of most genes expressed at a particular stage, and different isoforms are separated and transcript expression levels measured. Once determined by this technology, the expression profile of a gene must undergo validation by another, such as quantitative real-time PCR (qRT-PCR). This study aimed to select, annotate, and validate stress-inducible genes—and their promoters—differentially expressed in the leaves of oil palm (*Elaeis guineensis*) plants under saline stress.

**Results:**

The transcriptome analysis led to the selection of 14 genes that underwent structural and functional annotation, besides having their expression validated using the qRT-PCR technique. When compared, the RNA-Seq and qRT-PCR profiles of those genes resulted in some inconsistencies. The structural and functional annotation analysis of proteins coded by the selected genes showed that some of them are orthologs of genes reported as conferring resistance to salinity in other species. There were those coding for proteins related to the transport of salt into and out of cells, transcriptional regulatory activity, and opening and closing of stomata. The annotation analysis performed on the promoter sequence revealed 22 distinct types of cis-acting elements, and 14 of them are known to be involved in abiotic stress.

**Conclusion:**

This study has helped validate the process of an accurate selection of genes responsive to salt stress with a specific and predefined expression profile and their promoter sequence. Its results also can be used in molecular-genetics-assisted breeding programs. In addition, using the identified genes is a window of opportunity for strategies trying to relieve the damages arising from the salt stress in many glycophyte crops with economic importance.

## Background

Population growth and climate change affect the biomass production system on a regional, national, and global scale [1, 2]. The balance between demand and supply of food, fiber, feed, and bioenergy faces challenges of complex magnitude, demanding strong responses on several fronts—from scientists, policy analysts, and politicians, to mention a few [3]. Science is on the run to develop the necessary knowledge and technologies to guarantee this balance sustainably.

The molecular mechanisms of salt resistance were the object of extensive studies in Arabidopsis and agronomic plant species such as rice [4, 5, 6]. Salt stress directly alters biological and chemical compounds in plant cells, which activates the cellular response in glycophytic plants [7–9]. Furthermore, salt stress leads to ionic, secondary, osmotic, and oxidative stresses, triggering multiple complex signaling pathways [7].

Soil salinity is one of the abiotic stresses that threaten agriculture the most. Recently, it has gained momentum as a factor limiting the achievement of the sustainability goals in the Sustainable Development Agenda. Salinity stress causes numerous morphological, physiological, and biochemical changes in plants. Plants must maintain a high-water status in the face of water limitation and ionic toxicity to grow in saline conditions. As a result of salt stress, secondary stresses such as oxidative burst can occur, in which the production and accumulation of active radicals result in the oxidation of proteins and lipids and, eventually, the death of cells and plants [10].

In the ion transport process, there are two mechanisms conferring salinity resistance. First, the control of the influx and efflux of sodium, potassium, and calcium ions in the plant through the cell membranes in the roots to maintain the ionic balance of the cell and reduce the osmotic stress effect; and secondly, the transport and storage of ions within plant tissue, especially within the cell vacuole, by ionic and tonoplast membrane pumps to eliminate the effects of ionic toxicity [11]. If a plant can absorb water and excrete salt, it can grow and survive in saline conditions [12], and, given the growing saline lands, it seems necessary to consider strategies to increase salinity resistance to strengthen the biomass production system.

Recent studies on abiotic stress-related gene expressions enabled strategies to improve stress resistance in molecular breeding [13–15]. Gene expression analysis is commonly applied to understand molecular regulatory mechanisms and identify genes in current molecular biology [16, 17]. Transcriptome analysis using RNA sequencing has become the most used approach to identify the genes and the mechanisms involved in resistance to abiotic stress, such as saline stress in non-model organisms [18]. RNA sequencing nowadays is a technology that utilizes the ability of NGS to obtain an overall picture of the presence and amount of RNA in a given time interval. With transcriptome analysis, most genes expressed in a particular scenario—under salinity stress, for instance—can be identified and their expression level measured [10, 19, 20].

At the end of the process, it allows the selection of candidate genes for validation and, most important, achieves the best pipeline to reduce false positives and increase gain in cost and time effectiveness [21]. That is possible from an experimental design that represents the biological process of interest and allows better use of the data, followed by a robust bioinformatics pipeline that uses the most appropriate software according to the design. Both are needed to influence the achievement of the biological response as it is and ultimately achieve a high correlation between RNA-seq and quantitative real-time PCR (qRT-PCR) results. Thus, the present study aims to select a profile-specific salt stress-inducible set of genes, and their promoters, differentially expressed in the leaves of oil palm (*Elaeis guineensis*) plants under saline stress. Furthermore, the selected genes underwent qRT-PCR analysis to validate the expression profile seen in the RNA-Seq analysis. At last, the selected genes and their promoters underwent functional annotation analysis.

## Results

### Selection of profile-specific salt stress-inducible genes

**Fig. 1:**
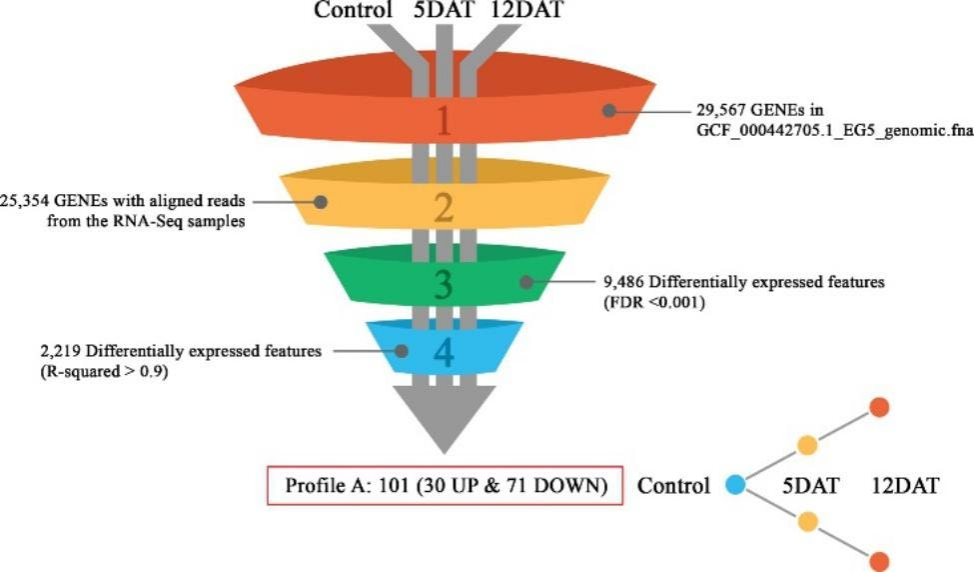
Strategy applied to prospect a profile-specific salt stress-inducible set of genes and their promoters differentially expressed at the leaves of oil palm (*Elaeis guineensis*) plants under saline stress

The strategy used to select salt stress-inducible genes bearing the desired expression profile (Profile A) resulted in 101 genes—being 30 upregulated twice [from control to 05 days after treatment (DAT), and again from 05 to 12 days], and 71 downregulated twice (Fig. 1), out of the 29,567 genes present in the reference oil palm genome [22]. The same strategy used to select salt stress-inducible proteins resulted in 21 upregulated and 49 downregulated ones out of the 43,551 proteins present in that genome. The first round of selection of differentially expressed genes used FDR (False Discovery Rate) ≤ 0.001 and R-squared ≥ 0.9.

The second round of selection, which prioritized the Log_2_(Fold Change) value (at 12 DAT in comparison with the control treatment), resulted in eight genes that upregulated from control to 05 days, and then again from 05 to 12 days, and six that downregulated twice. Those genes presenting the highest differences in expression level - in terms of fold change - went on to structural and functional annotation analysis.

### Expression analysis through qRT-PCR analysis

**Fig. 2:**
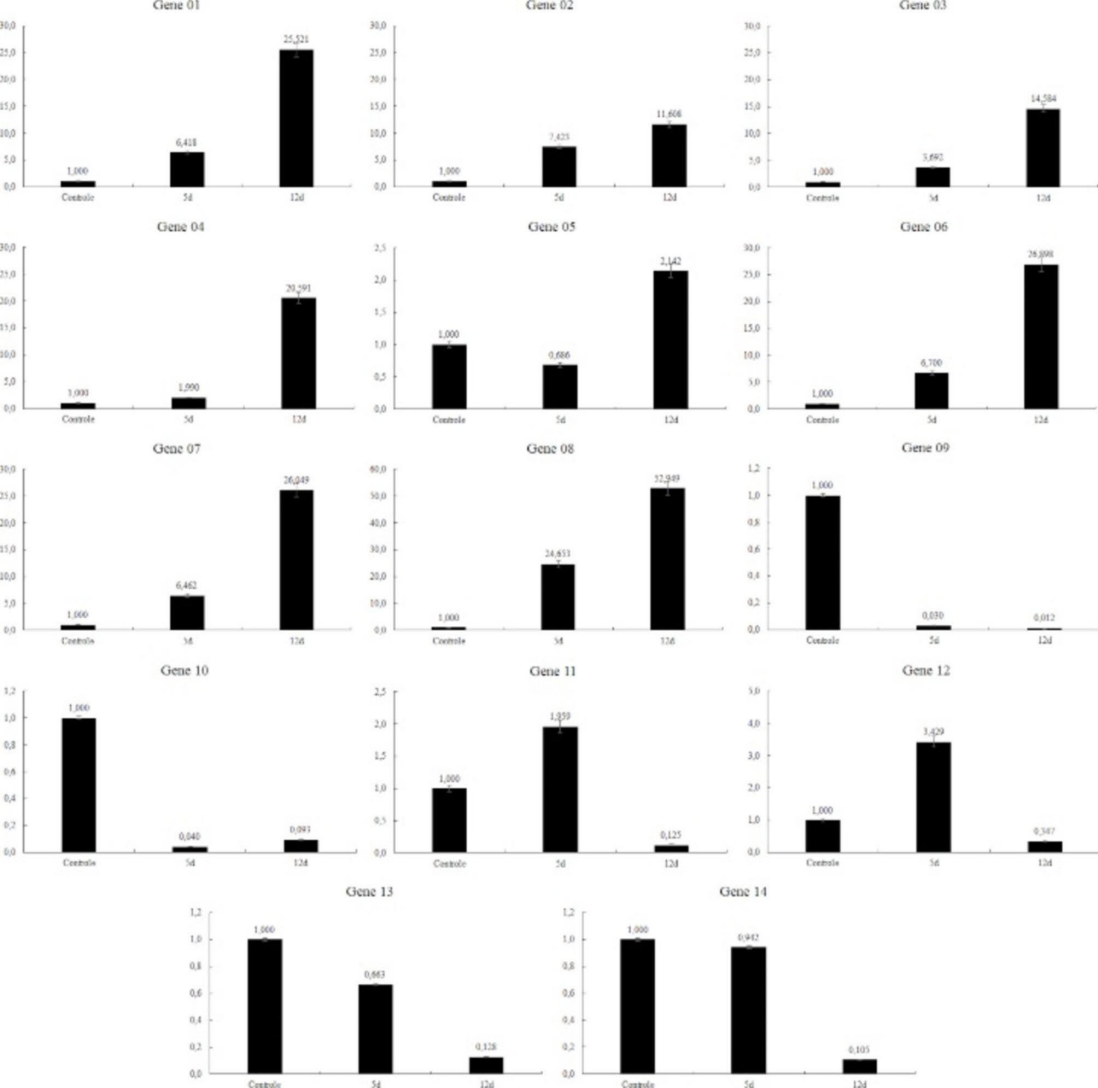
qRT-PCR analysis of the expression profile of the 14 salt-stressed-responsive genes selected in the genome of oil palm (*E. guineensis*). Internal control gene: EgEfMPOB00119.

The transcriptomics expression profile for each of the 14 selected genes is in Fig. 2. To compare control and stressed treatments, the expression level on the former was set up as 1.0, representing the initial transcription level. Among the genes tested, only genes 08, 11, and 12 presented an expression profile different than expected, in qualitative and quantitative terms—expression at 05 DAT was lower than at 12 DAT when upregulated or higher when downregulated.

**Fig. 3:**
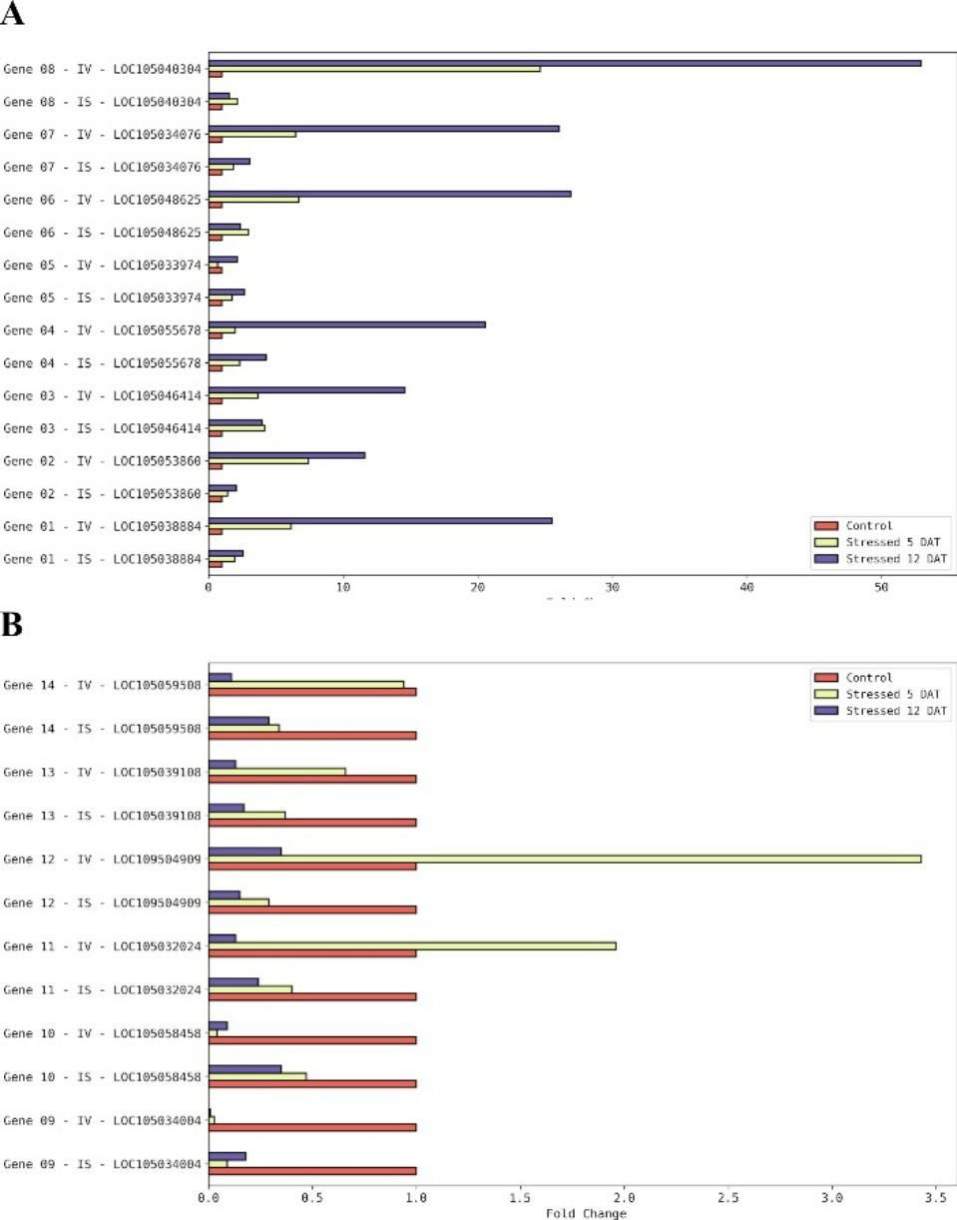
Comparison of the RNA-Seq (IS) and qRT-PCR (IV) differential expression analysis of the 14 salt-stressed-responsive genes selected in the genome of oil palm (*E. guineensis*). A—upregulated genes; B—downregulated genes

The quantitative differences between the RNA-Seq and qRT-PCR profiles are in Fig. 3. The upregulated genes showed expression levels at 12 DAT much higher for the latter than for the former, except for gene 05. These results corroborate the current knowledge that a strategy to select profile-specific stress-inducible genes and their promoters—for further use in molecular breeding—must not rely only on the RNA-Seq profile; the qRT-PCR validation is a complementary must.

### Structural and functional annotation of the coding region

**Fig. 4:**
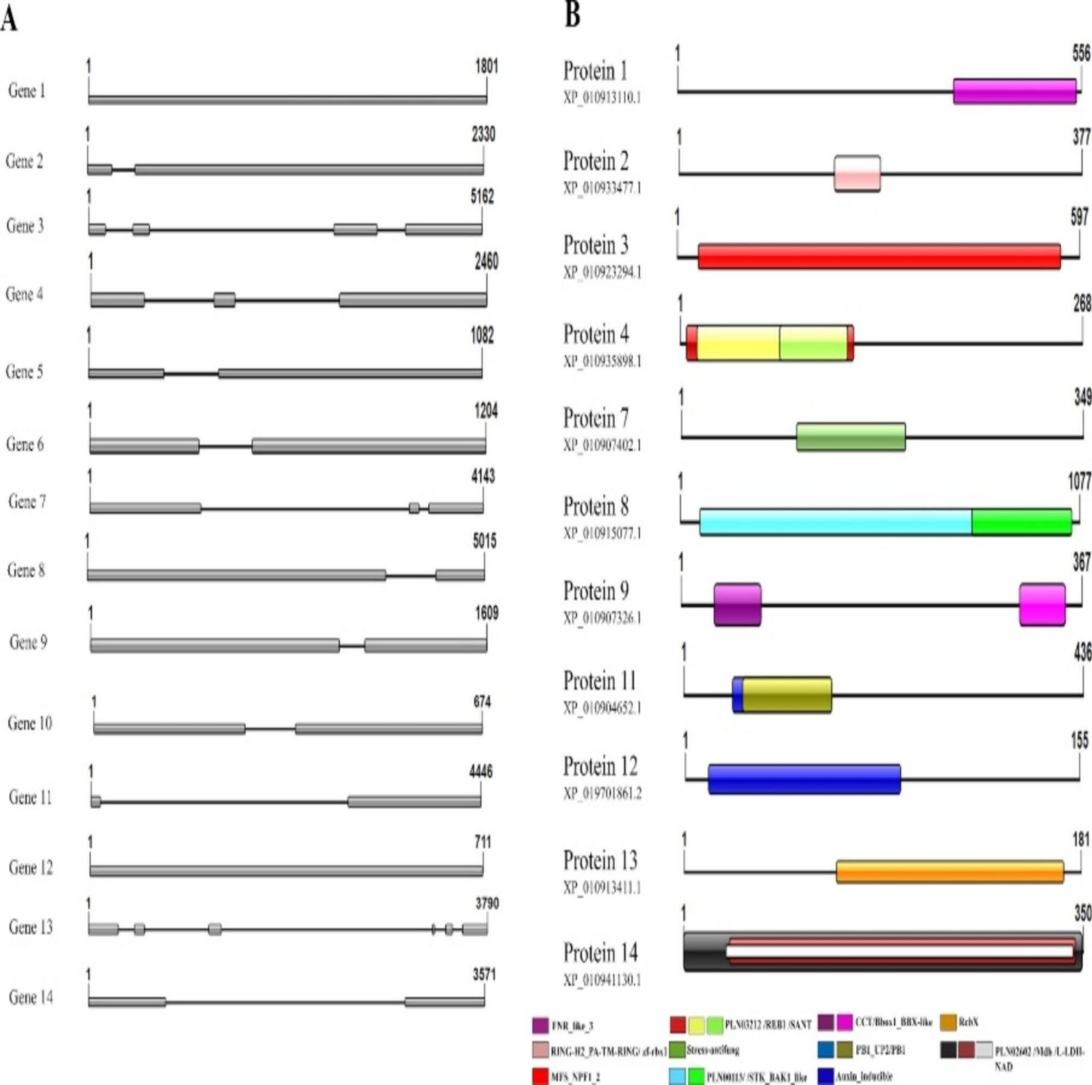
Structural annotation of the salt-stressed-responsive genes selected in the genome of oil palm (*E. guineensis*). A—Intron and exon number and location. The gray boxes represent the number of exons and their location in the gene; B—Domains of the 11 proteins that had NCBI platform hit. Each colored box represents a different domain and its location in the protein. The numbers in each row represent the total size of both genes and proteins

Regarding the coding region structure, those 14 genes showed diversity in the number of exons present. Genes 1 and 12 have one exon; genes 2, 5, 6, 9, 10, 11, and 14 have two; genes 4 and 7 have three; gene 3 has four, and gene 13 has six exons (Fig. 4 A). Eleven genes coded for proteins having known domains in their coding region. Eighteen distinct domain types appeared on those proteins (Fig. 4B). The proteins coded by genes 1, 2, 3, 7, 12, and 13 showed only one domain, while those by genes 8, 9, and 11 had two, and the ones coded by genes 4 and 14 presented three.

**Fig. 5:**
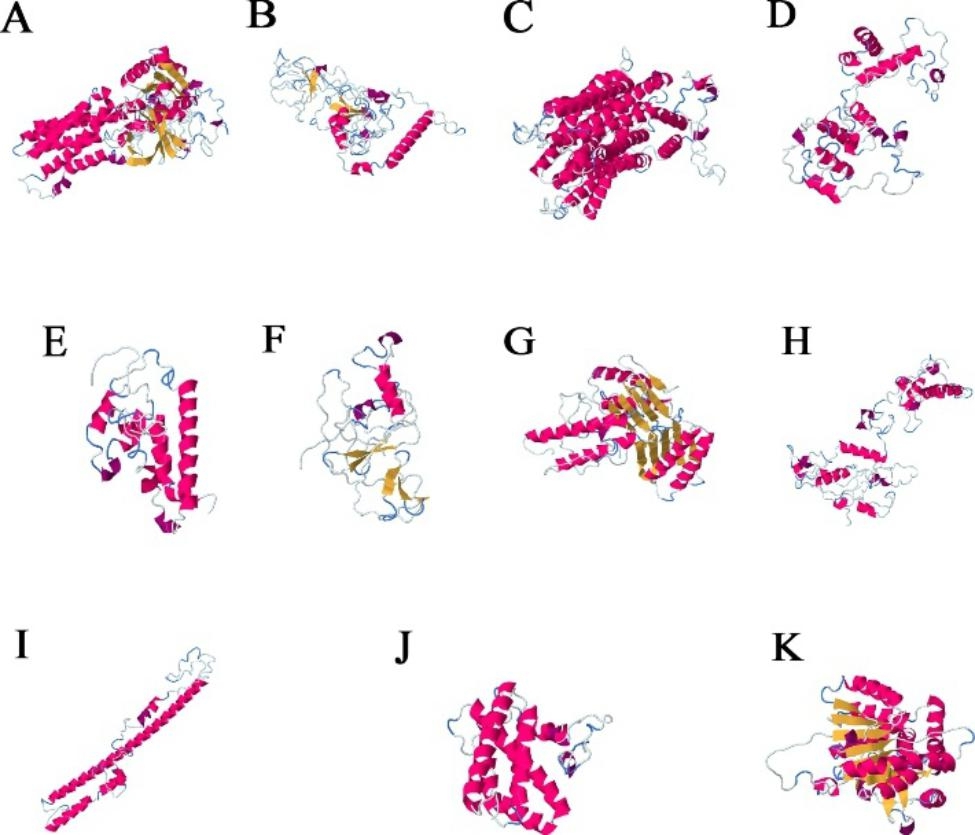
Tertiary structure of the proteins that had similarity with resolved structure of the PDB, obtained by the NCBI platform. A—Protein (XP_010913110.1) of gene 1; B—Protein (XP_010933477.1) of gene 2; C—Protein (XP_010923294.1) of gene 3; D—Protein (XP_010935898.1) of gene 4; E—Protein (XP_010907272.1) of gene 5; F—Protein (XP_010926289.1) of gene 6; G—Protein (XP_010907402.1) of gene 7; H—Protein (XP_010907326.1) of gene 9; I—Protein (XP_029124399.1) of gene 10; J—Protein (XP_010913411.1) of gene 13; K—Protein (XP_010941130.1) of gene 14. The models of proteins were obtained by the RaptorX online server. The α -helix, β -strand, and random coil are marked by red, yellow and blue, respectively

All selected proteins underwent modeling analysis by RaptorX to help understand their functional mechanism. The tertiary structures of 11 proteins are in Fig. 5, from genes 1, 2, 3, 4, 5, 6, 7, 9, 10, 13, and 14. According to the secondary structure also predicted by RaptorX, protein 1 presents the distribution of the helix class in most of its residues, followed by the coil class and beta class. The secondary structure of protein 2 has the distribution of the coil class in most residues, followed by the beta class and the helix class. As for proteins 3, 4, and 5, the helix is the class that stands out among the amino acid residues, followed by the coil and by beta. For protein 6, the coil class is more distributed among the residues, followed by beta and helix. The beta class stands out in protein 7, followed by coil and helix. For protein 9, the prevalent distribution is that of the coil class, followed by helix and beta. The amino acid residues for protein 10 are mainly of the helix class, followed by the coil and beta. The helix class was also predominant for proteins 13 and 14, followed by the coil class for protein 13 and beta for protein 14.

**Fig. 6:**
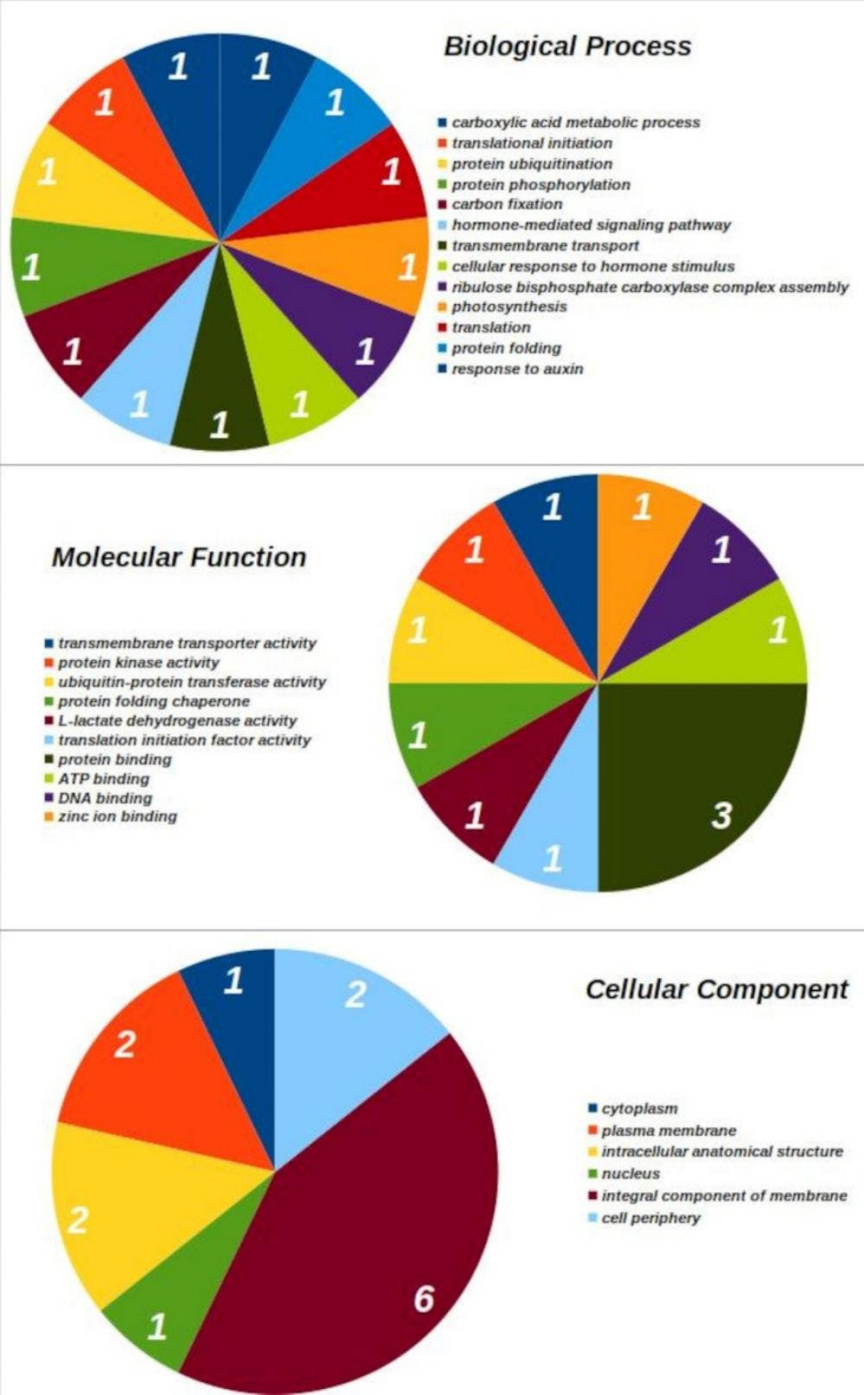
Gene Ontology (GO) annotation classification statistics graph from the salt-stressed-responsive genes selected in the genome of oil palm (*E. guineensis*); classified accordingly to biological process, cellular component, and molecular function. Numbers represent the amount of positive hits

After searching in the RaptorX software, according to the search for similarity with structures resolved with the PDB, it was possible to obtain several additional functional information of some of the proteins coded by the selected genes. The proteins that had similarities with tertiary structures resolved from the database were proteins 2, 3, 4, 7, 8, 9, and 13. The results of the structural and functional annotation for the 14 salt stress-inducible genes are in Table 1; Fig. 6. The proteins coded by those genes had positive hits to 13 biological processes, ten molecular functions, and six cellular components (Fig. 6). Protein binding (GO:0005515) was the molecular function with the higher number of positive hits, with three hits (Table 1). The InterProScan Search predicted that six of the 14 genes coded for an integral component of membranes (data not shown).

**Table 1:** Structural and functional characterization of the 14 stress-responsive genes prospected in the genome of oil palm (*Elaeis guineensis*)

SeqName	ID—NCBI	Description in NCBI	Chromosome or Scaffold Number	DNA Strand	Length (aa)	e-Value	sim mean	InterPro - Protein Family Membership	InterPro—GO terms	Enzyme Codes	Enzyme Names
Gene_01	LOC105038884	adenylate-forming reductase 06235-like	1	-	556	0.00E + 00	75.21	None predicted	No GO Terms	None	None
Gene_02	LOC105053860	E3 ubiquitin-protein ligase Os04g0590900-like	11	-	377	0.00E + 00	67.51	E3 ubiquitin-protein ligase RING1-like	No GO Terms	EC:2	Transferases
Gene_03	LOC105046414	protein NRT1/ PTR FAMILY 2.11-like	6	-	597	0.00E + 00	87.03	Proton-dependent oligopeptide transporter family	(GO:0055085)(GO:0022857)(GO:0016020)	EC:7	Translocases
Gene_04	LOC105055678	transcription factor MYB30-like	12	-	268	0.00E + 00	70.73	None predicted	No GO Terms	None	None
Gene_05	LOC105033974	uncharacterized protein LOC105033974	NW_011551084.1	+	149	4.01E-61	61.46	None predicted	No GO Terms	None	None
Gene_06	LOC105048625	putative mediator of RNA polymerase II transcription subunit 7	7	-	211	######	65.6	None predicted	No GO Terms	None	None
Gene_07	LOC105034076	plasmodesmata-located protein 7-like	NW_011551107.1	-	349	######	79.83	None predicted	No GO Terms	None	None
Gene_08	LOC105040304	probable LRR receptor-like serine/threonine-protein kinase At1g34110	3	+	1077	0.00E + 00	89.57	None predicted	(GO:0006468)(GO:0004672)(GO:0005524)(GO:0005515)	EC:2.7.1	Transferring phosphorus-containing groups
Gene_09	LOC105034004	zinc finger protein CONSTANS-LIKE 16-like	NW_011551090.1	-	367	0.00E + 00	59.34	None predicted	(GO:0008270)(GO:0005515)	None	None
Gene_10	LOC105058458	uncharacterized protein LOC105058458	15	-	195	######	65.8	None predicted	No GO Terms	None	None
Gene_11	LOC105032024	translation initiation factor IF-2	1	-	436	0.00E + 00	63.64	None predicted	(GO:0005515)	None	None
Gene_12	LOC109504909	auxin-responsive protein SAUR36-like	NW_011550957.1	+	155	######	78.74	Small auxin-up RNA	(GO:0009733)	None	None
Gene_13	LOC105039108	chaperonin-like RBCX protein 1, chloroplastic	2	-	181	######	86.79	Chaperonin-like RbcX	(GO:0110102)(GO:0044183)	None	None
Gene_14	LOC105059508	L-lactate dehydrogenase B-like	16	-	350	0.00E + 00	90.58	L-lactate/malate dehydrogenase	(GO:0019752)(GO:0016616)	EC:1.1.1.27	L-lactate dehydrogenase

### Structural and functional annotation of the promoter region

**Fig. 7:**
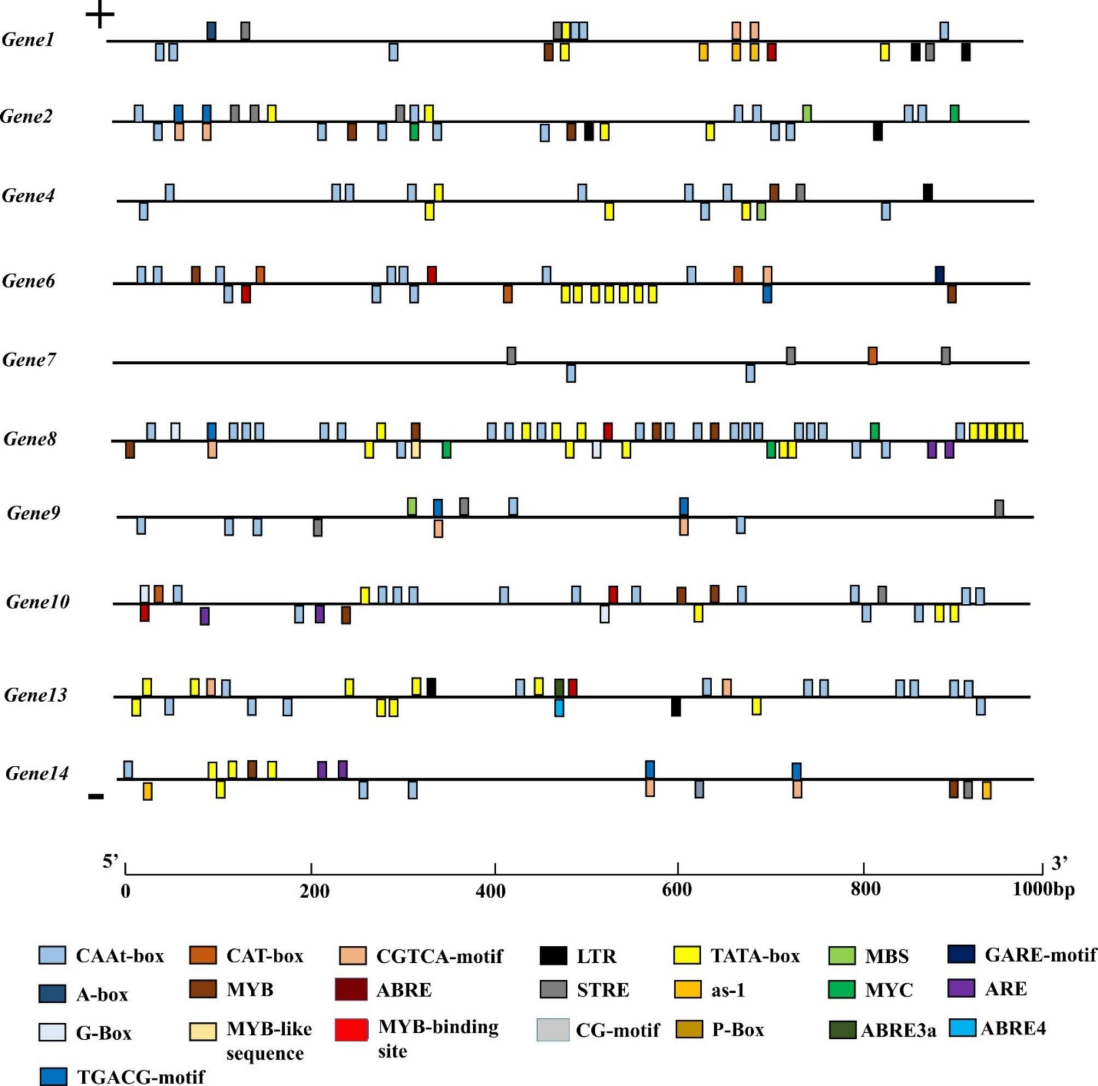
Cis-Acting elements located in the 1,000 bp long sequence upstream of the initiation codon of the salt-stressed-responsive genes selected in the genome of oil palm (*E. guineensis*). Note: ABRE was involved in the abscisic acid responsiveness; LTR involved in low-temperature responsiveness; G-Box-motif was involved in light responsiveness; CAAT-box was involved in promoter and enhancer regions; GARE-motif, P-box and TATA-box involved in gibberellin-responsiveness; CGTCA-motif and TGACG-motif were involved in the MeJA responsiveness; MBS was involved in drought-inducibility; ARE was involved in light responsiveness; CAT-box was involved in related to meristem expression; MYB was involved in drought-inducibility; MYC was a transcription factor response element; STRE was a non-biological absorption of related reaction elements

After submitting the putative promoter sequences—1,000 bp long sequence upstream of the initiation codon—of those 14 genes to PlantCARE to obtain cis-acting elements present in that region, it was possible to select ten promoters with positive hits in the PlantCare database. Twenty-two distinct cis-acting elements appeared in the promoter sequence of those genes (Fig. 7).

Eight out of the ten promoters presented the CAAT-box element, and seven had the MYB element, known to be related to abiotic stresses. Seven had the STRE cis-acting one, which undergoes activation by heat shock, low pH, lack of nutrients, and osmotic stresses. Six presented the ABRE and CGTCA, also related to abiotic stresses. Five showed the As-1, four showed the LTR and TGACG-motif elements, three the MBS and CAT-box elements related to abiotic stresses, and two the MYC and G-box elements related to transcription factors and different abiotic stresses. The cis-acting elements LTR, ABRE, ABRE 3a, ABRE4, G-Box, CAAT-box, CAT-box, MYB, MY-like sequence, MYB-binding site, STRE, TATA-box, MYC, and ARE, are found on the promoters of the selected genes and are linked to abiotic stresses [23, 24].

No pattern in the distribution of the cis-acting elements is evident when comparing the profile of these ten promoters evaluated, neither among those from upregulated nor downregulated ones. The 1,000 bp long sequence upstream of the initiation codon from genes 7 and 9 did not show the TATA-box element.

## Discussion

Among the few studies reporting on the expression profile of oil palm genes under salinity stress [23, 24], neither carried out a strategy to prospect and characterize stress-inducible genes and their promoters nor compared RNA-Seq and qRT-PCR expression profiles. Here we selected a profile-specific salt stress-inducible set of 14 genes and their promoter sequences by applying a time-course differential expression analysis. Eight genes were positively regulated twice, and six negatively regulated twice—initially from control to 05 DAT and later from 05 to 12 DAT. It is necessary to highlight the statistical criteria for selecting salt-responsive genes in oil palm. In the present study, we used a 99.9% reliability level and an R-squared ≥ 0.9, besides the fold change, to select such genes.

Research on gene expression mechanisms has contributed to unraveling the complex regulations in the life cycle of a plant, including how it responds to a saline environment. Transcription technology can reveal candidate genes and key pathways involved in salt resistance by analyzing differentially expressed genes and performing their functional annotation [25]. Additionally, qRT-PCR is the most applied technique to determine the expression level of a target gene. This technique is one of the most sensitive, accurate, and reproducible techniques to quantify the expression of specific genes. It has become the most common method for validating RNAseq results, requiring a normalization method against reference genes to achieve reliable results [26, 27].

The performance of absolute quantification (gene expression correlation between RNA-Seq and qRT-PCR data) without assessing the performance of relative quantification (correlation of differential gene expression) has been the usual way of analysis, and the latter is the goal of most RNA-seq studies [26, 27]. Feng and colleagues [21] developed a series of performance parameters to evaluate RNA-seq quantification workflows when performing analyses in celery under abiotic stress and hormone treatment. Regarding the qualitative comparison of the expected expression profile for each gene selected in the present study, most genes presented the expression profile in the qRT-PCR analysis similar to the RNA-Seq differential expression study. On the other hand, genes 5, 11, and 12 did not repeat the RNA-Seq expression profile. That may be due to a version of a reference genome still needing improvement and an RNA-Seq experimental design with only three replicates per treatment. When analyzing the quantitative side of the comparison, some differences are evident. The increase in expression of genes 1, 4, 6, and 8 in RNA-Seq was lower than fivefold, while in qRT-PCR, it ranged from 20 to 50. Genes 10, 13, and 14 expressions dropped approximately 50–60% in the RNA-Seq analysis, and the qRT-PCR analysis showed a drop of about 90%.

This set of genes is *per se* a source of candidate genes to undergo future validation of their capacity to confer resistance to salinity stress via heterologous expression in model plants. Once this role is confirmed, a subsequent step would be the horizontal transfer (or gene editing) to economically important glycophyte plants [28]. For instance, gene 2, an E3 ubiquitin-protein ligase Os04g0590900 gene, came out as a member of the E3 ubiquitin-protein ligase RING1-like family. Many E3 ligase targets are proteins involved in abiotic stress responses, such as salt. Several E3 ligases regulate these responses to salinity stress by targeting and mediating the degradation of salt stress-related proteins [29]. Gene 4, a transcription factor MYB30, is another example. This gene codes for a protein that came out as one with family membership non-predicted and classified as an MYB family transcription factor in the Panther Classification System. MYB30 modulates plant salt resistance through the positive regulation of mitochondrial alternative oxidase AOX1a, and MYB30 mutants exhibit hypersensitivity to salt stress [30].

The remaining proteins selected in the present study stand out for their several distinct functions, found out by similarity in the PDB analysis, ranging from membrane proteins that may be related to the transport of salt into and out of cells, transport of ions, transcription related to transcriptional regulatory activity, and opening and closing of stomata. Some showed trans-membrane, cytoplasmic, and(or) non-cytoplasmic domains predicted (genes 1, 2, 3, 5, 7, and 10).

The α-helices, predominant in the proteins coded by the genes selected in this study, can serve as anchors to the lipid bilayer or form a channel through which various substances can pass. In the latter case, the α-helices have hydrophilic residues directed toward a channel and hydrophobic residues directed away from it and interacting with the lipid bilayer. In this way, many polar substances that, in the absence of proteins, could not cross the membrane will be able to do so through these channels [31]. The protein from gene 3 has similarities to the crystal structure of the nitrate transporter NRT1.1, a transport and membrane protein from *A. thaliana*, which molecular functions are related to the transmembrane activity [32]. The response to nitrate and different stimuli, such as salt stress—stand out among the biological processes [33]. The salt level linked to the low ability to exclude salt causes marked damage in older leaves of glycophyte plants. Such damage occurs due to the accumulation of ions and anions being superior to the compartmentalization capacity in the vacuoles of the cells, leading to cell death from salt intoxication or dehydration, depending on where such ions have accumulated—cytoplasm or cell wall. Transport and membrane proteins are critical to removing toxins inside cells [34, 35].

The protein coded by gene 13 has an affinity with the AtRbcX1 structure of *A. thaliana*, which is a chaperone. AtRbcX1 molecular function is protein folding, its biological process involves metabolic processes and carbon fixation, and its cellular component encompasses intracellular anatomical structure, chloroplast, stomatal complex, thylakoid, and plastid. A response commonly observed in plants under saline stress is stomatal closure, and in this condition, the amount of carbon dioxide gets compromised, inhibiting carbon fixation. Chloroplasts, in turn, are exposed to excess energy, which increases the generation of reactive oxygen species [36].

The number and shape of cis-elements in promoter regions can play an essential role in regulating gene expression related to different metabolic pathways [37–42]. Therefore, the 1000 bp upstream region of genes with similar expression profiles were subjected to a similarity search for the cis-acting elements in common among them. Genes with STRE, MYB, MYC, and MYB-binding site cis-acting elements in their promoters showed increased expression when subjected to salt stress. Finally, regarding downregulated genes, when the gene has the promoter with cis-acting elements ABRE4, ARE, and MYB-like in the upstream region of the start codon, it experiences a reduction of expression when subjected to saline stress [38].

In the present study, among the cis-elements found in the promoter regions of the 14 genes, the MYC, G-Box, ABRE, and TATA-box are linked to salinity [37, 39, 42]. Although there are reports on TATA-less promoters in plant genomes [43–45], this not usual result seen in the promoter sequences from genes 7 and 9 can also be a result of the quality of the reference genome used. Further analysis is necessary to determine whether it is a case of the former or the latter.

Once looking for a specific distribution pattern of cis-acting elements in the putative promoter region, the present results confirm that it will be necessary to have many more sequences to find and consequently design markers for *in silico* genome-wide search for salinity-responsive genes. However, as it is known, one can select some of these cis-acting elements to target in an editing strategy aiming to interfere with the translation and, consequently, the expression of one or more proteins, which could change their function concerning saline stress. Therefore, they would become a target site for CRISPR-based testing for exact modifications in the cis-acting sequence, either to silence the gene or to place it in an upstream region of a not salt-responsive gene and then analyze the effect of that. The novel CRISPR–Cas9 genome editing system will be a factor in achieving a more precise horizontal transfer, reducing, to a certain extent, the need for some biosafety analysis demanded today for genetically modified plants [46].

The current study is another step in our work on characterizing the morphophysiological and molecular responses of oil palm plants to salinity stress [47–50]. Besides developing a salinization protocol successful in generating different levels of stress by gradients of electrical conductivity and water potential [47], the studies that followed up led to the identification of salt-responsive miRNAs and their putative target genes [48], as well as mapping the main pathways affected by this abiotic stress [49]. On top of that, they allowed the identification of several salt-responsive genes, proteins, metabolites, and promoter sequences [48, 49, 50, and the present report] which might open windows of opportunity to develop salt-tolerant oil palm genotypes via genetic engineering/editing approaches.

## Conclusion

Our study reports a strategy to select profile-specific salt stress-inducible genes and their promoters. This strategy employed RNA-Seq followed by time-course differential expression analysis. In addition, a quantitative and qualitative comparison study tried to validate the RNA-Seq results using qRT-PCR. Fourteen differentially expressed genes were selected and validated in this study. Some inconsistencies did appear when comparing the expression profiles—RNA-seq against qRT-PCR—that may have to do with the difference in sensitivity between them and the amount of biological and technical replicates used. This set of genes is *per se* a source of candidate genes to undergo future validation of their capacity to confer resistance to salinity stress. Regarding the promoters and their gene regulation regions, it was not yet possible to infer that a specific combination of cis-acting elements is a good candidate marker for future use in a genome-wide search for this type of profile-specific salt stress-inducible genes. However, one can consider some of the cis-acting seen as a target for gene editing by CRISPR–Cas9 technology.

## Materials and methods

### Oil palm transcriptome database

All oil palm plants used in the study were derived from embryogenic callus from genotype AM33, a Deli x Ghana from ASD Costa Rica (http://www.asd-cr.com). The AM33 genotype is a plant from a commercial field in the State of Pará, Brazil. Prof. Sergio Motoike, from the Universidade Federal de Viçosa—UFV, located in Minas Gerais, Brazil, supplied the embryogenic calluses from AM33. Young oil palm plants at the growth stage known as “bifid” seedlings were subjected to two different doses of NaCl in March 2018 and maintained under these conditions for 12 days in a completely randomized experimental design in a greenhouse in Brasília, DF, Brazil (15,732 ° S, 47,900° W, 1,030 m of altitude) [47].

The decision on which samples for transcriptomics analysis to use came after taking into consideration the morphophysiological responses of the young oil palm plants to salinity stress. Vieira et al. [47] characterized those responses by submitting the plants to different treatments (0.0, 0.5, 1.0, 1.5, and 2.0 g NaCl per 100 g substrate on a dry basis), with five replicates per treatment. For the present study, we collected the apical leaves from three stressed plants (2.0 g NaCl, or ~ 40 dS m^− 1^ of electrical conductivity) at 05 and 12 days after the stress onset (DAT), together with the apical leaves from three control plants at 12 days (0.0 g NaCl, or ~ 2 dS m^− 1^), immediately frozen in liquid nitrogen and stored at − 80 °C until RNA-Seq. More details about the plant material, growth conditions, saline stress conditions, and the experimental design used in the study that generated this database were previously reported [47–50].

### RNA-Seq data analysis

The raw data analyzed in this study are available in the Sequence Read Archive (SRA) database of the National Center for Biotechnology Information—BioProject PRJNA573093, BioSample SAMN12799239. The OmicsBox Bioinformatics Platform [50] was employed to perform all RNA-Seq analyses, as described previously [48–50]. To run the time-course expression analysis among the treatments, we used the default parameters based on the software package maSigPro, from to the Bioconductor project, FDR (False Discovery Rate) ≤ 0.001, R-squared ≥ 0.9, without the use of a filter for low counts genes, and using the Trimmed mean of M values method of normalization [51]. Each treatment was represented in this RNA-Seq study by three replicates - or three plants.

### RNA extraction, reverse transcriptase-PCR and quantitative real-time PCR analyses

After being collected from young oil palm plants at 5 (stressed) and 12 (control and stressed) DAT, the apical leaves underwent immersion in liquid nitrogen and then stored at -80 °C until RNA extraction. Total RNA was isolated using the Qiagen Rneasy® Plant Mini kit (QIAGEN, CA, USA) following the manufacturer’s protocol, and RNA quantity was measured using the Nanodrop Qubit 2.0 Fluorometer (Life Technologies, CA, USA). After that, the extracted RNA was used as a template for reverse transcription to obtain cDNA using the commercial kit SYBR® GreenER™ qPCR SuperMix Universal (Invitrogen®). The gene named EgEfMPOB00119 40 S ribosomal protein S23 mRNA, complete cds, mRNA sequence present in *E. guineensis*, was chosen as a positive control (constitutive gene).

The mRNA fasta files from the selected genes were downloaded from NCBI and exported to the PerlPrimer software [52] for designing the primers (Table 2). The qRT-PCRs were carried out in optical 96-well plats in a 7500 Real-Time PCR System (Applied Biosystems, Foster City, CA, USA) with an SYBR® GreenER™ qPCR SuperMix Universal (Invitrogen®) (INVITROGEN, 2010), following the manufacturer’s instructions. The thermal cycler was set as follows: (a) 95ºC for 34 s, 95ºC for 5 s, 60ºC for 34 s for 40 cycles, and at the second step of each cycle, fluorescence was obtained; (b) the dissolution curve was acquired as followed: 95ºC for 15s, 60ºC for 60s and 95ºC for 15s. Fluorescence readings were performed by StepOnePlus™ Real-Time PCR at each amplification cycle and, later, analyzed by StepOnePlus™ software v2.3—Life Technologies. Two biological replicates and three technical replicates were used for this study, generating six reads per gene per treatment. The method of 2 − ∆∆CT was adopted to represent relative expression levels of the genes [26].

**Table 2:** Pairs of primers designed and used for differential expression analysis of the 14 prospected genes by means of the qPCR technique

Gene	5’- end Primer	3’- end Primer
GENE_01	CTTCCAAGCCGAAACAACC	TACACCGTGAACAGTCCCT
GENE_02	AGAGCCAGTTGCTATCTCC	ATACTTGATGGCAGTGGAAGG
GENE_03	TCAATTCAGGCTTGTTGAC	GAATGCGATCATAAACAGGT
GENE_04	CAAGCAAGCTGGTCTATTGAG	TTCCTCCGAAGAATCCGTG
GENE_05	ATCTCCTCTGAGGAGAGG	AGACAGCAAGAGCAACAG
GENE_06	AATCCACCATCAGATGCACAG	ATCTGGCCTGACTTGCCA
GENE_07	AGACACGATCACGTTGATGAG	GAGAAGAGCATAATGGACAACAC
GENE_08	TTGCACCAGAGTATGGTTACAC	AAATCTACCACCTTCAGGGAC
GENE_09	GACAACGCTATCACCTACACC	TCTCCCTATACCTCGTCACCT
GENE_10	ACTTATCCAATCGCACCT	TAATGCAATACCCACTCCAC
GENE_11	CTTCTGCCAATATGTATTCCTCC	CTGGTACTTGATGGAGAGTAGG
GENE_12	TAGTCACCACCAACCTCAG	GAGCCTTGTCCATATCCACAG
GENE_13	CGCAACTTGAGAGCTATAATCC	GTATACTCGTATGAAGGTGGTG
GENE_14	ATGTGCAGGCCTACATGG	GGTTAGACTCCTCATCTGTGAG
EgEfMPOB00119*	CCAGGGTTCAGCTGATTAAG	TCGTCCAAATCCAGCAATC

* Internal Control Gene

### Structural & functional annotation

For the structural annotation of the sequences of the chosen genes, the symbol, description, location on chromosome, number of exons, type, and direction of each gene underwent analysis using the information available in the NCBI. The search for functional domains in the proteins used the NCBI Conserved Domain Search (www.ncbi.nlm.nih.gov/Structure/cdd/wrpsb.cgi). Parameter settings influencing the query execution results were the following: E-value ≤ 1. With this, it was possible to produce the images by the IBS Illustrator Software [53]. The Software RaptorX ( http://raptorx.uchicago.edu/ContactMap) was used to investigate the tertiary structure of the proteins. In addition to the search for tertiary structures of proteins, a BLASTp was performed with the protein sequences in the BLAST database (Basic Local Alignment Search Tool) to search for similarity with resolved structures in the PDB.

Based on the oil palm reference genome [22], 1,000 bp upstream sequences of the selected genes were acquired. PlantCARE was used to analyze them with the default parameter (http://bioinformatics.psb.ugent.be/webtools/plantcare/html) [54]. Parameter settings were the following: matrix score less than or equal to 5. The IBS Illustrator Software was again used to produce the images [53].

## Data Availability

All RNA-seq fastq files used in this study have been uploaded in the SRA database of the NCBI under *Elaeis guineensis* Transcriptome_Drought and Salinity Stresses - BioProject PRJNA573093 (SUB6324604), BioSample SAMN12799239 (SUB6325749), SRA submission SUB6335775 (accessions from SRR10219424 to SRR10219441). The data-sets used and/or analyzed during the current study are available from the corresponding author on reasonable request.

## Declarations

### Author information

^1^Graduate Program of Plant Biotechnology, Federal University of Lavras, CP 3037, Lavras, MG, Zip Code 37200-000, Brazil. ^2^Brazilian Agricultural Research Corporation, Embrapa Agroenergy, Brasília, DF, Zip Code 70770-901, Brazil. ^3^ Brazilian Agricultural Research Corporation, Embrapa Mid-North, Teresina, PI, Zip Code 64008-780, Brazil.

## Abbreviations

CRISPR: Clustered Regularly Interspaced Short Palindromic Repeats
DAT: days after imposition of the treatments
FDR: False Discovery Rate
GO: Gene Ontology
NCBI: National Center for Biotechnology Information
NGS: Next-Generation Sequencing
PDB: Protein Database
IBS: Illustrator for Biological Sequences
qPCR: quantitive PCR
